# Weather conditions determine attenuation and speed of sound: Environmental limitations for monitoring and analyzing bat echolocation

**DOI:** 10.1002/ece3.4088

**Published:** 2018-04-24

**Authors:** Holger R. Goerlitz

**Affiliations:** ^1^ Acoustic and Functional Ecology Group Max Planck Institute for Ornithology Seewiesen Germany

**Keywords:** acoustic tracking, automatic species identification, call analysis, Chiroptera, environmental variation, ranging accuracy

## Abstract

Echolocating bats are regularly studied to investigate auditory‐guided behaviors and as important bioindicators. Bioacoustic monitoring methods based on echolocation calls are increasingly used for risk assessment and to ultimately inform conservation strategies for bats. As echolocation calls transmit through the air at the speed of sound, they undergo changes due to atmospheric and geometric attenuation. Both the speed of sound and atmospheric attenuation, however, are variable and determined by weather conditions, particularly temperature and relative humidity. Changing weather conditions thus cause variation in analyzed call parameters, limiting our ability to detect, and correctly analyze bat calls. Here, I use real‐world weather data to exemplify the effect of varying weather conditions on the acoustic properties of air. I then present atmospheric attenuation and speed of sound for the global range of weather conditions and bat call frequencies to show their relative effects. Atmospheric attenuation is a nonlinear function of call frequency, temperature, relative humidity, and atmospheric pressure. While atmospheric attenuation is strongly positively correlated with call frequency, it is also significantly influenced by temperature and relative humidity in a complex nonlinear fashion. Variable weather conditions thus result in variable and unknown effects on the recorded call, affecting estimates of call frequency and intensity, particularly for high frequencies. Weather‐induced variation in speed of sound reaches up to about ±3%, but is generally much smaller and only relevant for acoustic localization methods of bats. The frequency‐ and weather‐dependent variation in atmospheric attenuation has a threefold effect on bioacoustic monitoring of bats: It limits our capability (1) to monitor bats equally across time, space, and species, (2) to correctly measure frequency parameters of bat echolocation calls, particularly for high frequencies, and (3) to correctly identify bat species in species‐rich assemblies or for sympatric species with similar call designs.

## 1 INTRODUCTION

Bioacoustic research is the study of animal sounds, their production, transmission and perception (Bradbury & Vehrencamp, 2011; Fletcher, 2007) and regularly involves the recording and acoustic analysis of animal sounds. The characteristics of a recorded sound are generally determined by (1) the sound source (e.g., the animal), (2) the sound transmission channel (e.g., the animal's environment), and (3) the recording equipment (i.e., microphones). While we are interested in the animal and its sounds, we need to take into account that the transmission channel and the recording equipment can significantly affect the measured sound parameters. On land, the air forms the sound transmission channel through which sound propagates. As air conditions such as temperature and humidity are not constant, but vary over space and time (i.e., weather), the properties of the sound transmission channel also vary (e.g., Marten & Marler, 1977; Marten, Quine, & Marler, 1977; Wiley & Richards, 1978). This paper addresses the influence of the weather, that is, environmental variation in atmospheric conditions, as a ubiquitous source of error on measured sound parameters, particularly for high‐frequency sounds such as bat echolocation calls.

Echolocating bats are auditory specialists, and are therefore regularly studied by researchers as champion species to understand auditory‐guided behavior (e.g., Denzinger & Schnitzler, 2013; Moss & Surlykke, 2001; Thomas, Moss, & Vater, 2004). Bats emit calls of usually high ultrasonic frequencies, which transmit through the air, are reflected off environmental objects and transmit back as echoes to the bat, enabling the bat to perceive object properties (e.g., Goerlitz, Geberl, & Wiegrebe, 2010; Goerlitz, Genzel, & Wiegrebe, 2012; Surlykke, Nachtigall, Fay, & Popper, 2014). In addition, bats are becoming increasingly important as bioindicators (Jones, Jacobs, Kunz, Willig, & Racey, 2009). Many bats use echolocation, an active sensory system (Nelson & MacIver, 2006), as main remote sense for perceiving their environment. Researchers take advantage of this continuous stream of echolocation calls to acoustically monitor bat presence/absence, activity and behavior and, in certain cases, to identify species (Andreassen, Surlykke, & Hallam, 2014; Walters et al., 2012; Zamora‐Gutierrez et al., 2016). Beyond academic research, these data are used for risk assessment (e.g., at wind turbines; e.g., Newson et al., 2017), to infer population density, diversity, and vulnerability of bats (Clement, Rodhouse, Ormsbee, Szewczak, & Nichols, 2014; Meyer et al., 2011), and to inform risk mitigation and conservation strategies (Meyer, 2015), often based on automatic call analysis software (Russo & Voigt, 2016; Rydell, Nyman, Eklöf, Jones, & Russo, 2017). While these acoustic methods became increasingly easier, faster and more powerful, many biological, environmental, and technical factors lead to variation, if not errors, in the results (Adams, Jantzen, Hamilton, & Fenton, 2012; Brumm, Zollinger, Niemela, & Sprau, 2017; Rydell et al., 2017; Zollinger, Podos, Nemeth, Goller, & Brumm, 2012). Understanding these factors is therefore paramount for correct bioacoustic measurements.

Here, I concentrate on two main features of the transmission channel, namely atmospheric attenuation and speed of sound, which are highly relevant when analyzing sounds, both as a bat and as a bioacoustic researcher. Atmospheric attenuation (AA) is the reduction in sound amplitude while the sound travels through the air, caused by absorption of acoustic energy by the air molecules (Attenborough, 2007). AA is determined by four factors: sound frequency, air temperature, relative humidity, and pressure (Attenborough, 2007). Crucially, these factors interact in a complex, nonlinear fashion, meaning that no simple relationship exists between AA and weather conditions (Attenborough, 2007; Griffin, 1971; Luo, Koselj, Zsebők, Siemers, & Goerlitz, 2013; Stilz & Schnitzler, 2012). The magnitude of AA determines the detection range of sound, which varies with sound frequency and weather conditions. This causes differential detection ranges for different call frequencies and for different weather conditions, which may limit the analysis of call frequency parameters and the detection and identification of bat species. Likewise, this weather‐induced variation in AA is also significant for the bats, as supported by the correlation between geographic and local differences in environmental conditions and bat call frequencies. For example, populations of several horseshoe bat species (Jacobs, Catto, Mutumi, Finger, & Webala, 2017; Maluleke, Jacobs, & Winker, 2017; Mutumi, Jacobs, & Winker, 2016) and *Hipposideros ruber* (Guillen, Juste, & Ibanez, 2000) exhibit geographic differences in call frequency correlated with average weather conditions, suggesting adaptation to average weather conditions over evolutionary time. Importantly, however, also bats whose echolocation is adapted to average weather conditions experience additional spatial and temporal fluctuations in weather conditions and AA, leading to variation in call and echo detection range. Some bats seem to adapt their calls even to current local weather conditions. Chaverri and Quiros (2017) showed weather‐frequency‐correlation in the low‐frequency call type of two neotropical free‐tailed bats, suggesting short‐term individual behavioral flexibility in calls optimized for long‐range detection.

The speed of sound (*c*) is the speed by which sounds propagate through the air (often approximated by 340 m/s). It is the fundamental physical sound parameter by which bats compute object range based on echo delay, as well as the fundamental parameter underlying the acoustic localization of echolocating bats and other sound‐producing animals (reviewed in Blumstein et al., 2011), a method that is increasingly used to obtain bats' spatial positions based on the differences in arrival time of the same call on multiple microphones (e.g., Fujioka, Aihara, Sumiya, Aihara, & Hiryu, 2016; Goerlitz, ter Hofstede, Zeale, Jones, & Holderied, 2010; Hügel et al., 2017; Lewanzik & Goerlitz, 2018; Seibert, Koblitz, Denzinger, & Schnitzler, 2013; Surlykke, Pedersen, & Jakobsen, 2009).

Although the physical relationships between atmospheric conditions and AA (Bass, Sutherland, Zuckerwar, Blackstock, & Hester, 1995; Bazley, 1976; Evans, Bass, & Sutherland, 1972; Giacomo, 1982) and *c* (e.g., Cramer, 1993; Wong, 1986) are described, these technical papers are not necessarily read by ecologists and bioacousticians (but see these bat‐specific articles: Griffin, 1971; Hartley, 1989; Lawrence & Simmons, 1982; Stilz & Schnitzler, 2012). The objectives of this paper thus are to highlight and summarize the effects of the atmosphere on sound recordings and analysis. Specific objectives are (1) to illustrate the variation in atmospheric attenuation and speed of sound as a function of weather conditions, (2) to show how this variation influences the measurement of acoustic signals, and (3) to discuss how this may cause species‐specific limits in call analysis, bat species identification and abundance measures. To achieve these objectives, I aim to provide an accessible overview of the atmospheric effects on AA and *c*, provide the methods for their calculations in the SI as Matlab, Python, R and Excel code, and discuss their influences on bioacoustic measurements.

## 2 METHODS

### 2.1 Calculation of atmospheric attenuation and speed of sound

Atmospheric attenuation (AA, in dB/m) was calculated as function of sound frequency (*f*, in Hz) and the temperature (*T*, in °C), relative humidity (RH, in %), and pressure (*p*, in Pa) of the air (ISO standard #9613‐1, ISO 1993). Speed of sound (*c*, in m/s) was calculated as function of temperature, relative humidity and pressure of the air, and the molar fraction of CO_2_ in air (Cramer, 1993). All equations are available in the [Link], [Link] (as text, as Matlab, Python and R code, and as Excel spreadsheets).

Calculations were restricted to sound frequencies and atmospheric conditions most relevant for echolocating bats. Particularly, I used sound frequencies from 10 to 110 kHz (in steps of 1 kHz), focusing on the dominant call frequencies for many bat species (Safi & Siemers, 2010; Schnitzler, Moss, & Denzinger, 2003). Temperature ranged from 5 to 40°C (step size 1°C), relative humidity from 0% to 100% (step size 1%), and atmospheric pressure from 670 to 1,070 hPa (step size 10 hPa; corresponding to altitudes of approximately 3,300 m, the maximum flight height of *Tadarida brasiliensis* (Williams, Ireland, & Williams, 1973) to −425 m, the lowest point on Earth at the Dead Sea). The molar fraction of CO_2_ was set to approximate current levels of 400 ppm (IPCC, 2013).

### 2.2 Exemplary weather data

In addition to using the full range of relevant atmospheric parameters, I also calculated AA and *c* for two sets of exemplary real weather data to show realistic temporal and spatial variation. To show exemplary daily fluctuations in AA and *c*, I used weather station data (*T,* RH) from July 2016 in Konstanz, South Germany collected daily at 10 pm (i.e., within the first hour after sunset). *p* was set to the local average in July 2016 of 1,020 hPa, and the molar fraction of CO_2_ to 400 ppm. A second large‐scale set of weather data (*T*, RH) over three years during bat peak activity was downloaded from Weather Online UK (http://www.weatheronline.co.uk; cf. Luo et al., 2013) for four areas with different climatic conditions (Germany, Malaysia, Negev desert, South Africa). Here, *p* was set to 1013.25 hPa and the molar fraction of CO_2_ of 400 ppm.

### 2.3 Estimating localization error

Acoustic localization methods convert time of arrival differences (TOAD) of the same call at multiple microphones into 3D‐positions based on speed of sound *c*, and thus are susceptible to variation in the speed of sound. To estimate the localization error caused by errors in *c*, I generated the call sequences that a microphone array would receive from a bat at different spatial positions, and then analyzed these sequences with a different *c* than used during generation. Specifically, I used cross‐correlation to calculated TOADs for symmetrical planar star‐shaped four‐microphone arrays with 60 cm (e.g., Goerlitz, ter Hofstede et al., 2010; Hügel et al., 2017; Lewanzik & Goerlitz, 2018) and 120 cm intermicrophone distance, a *c* of 338 m/s, and a grid (2 m spacing) of bat positions filling half a hemisphere above the array up to 20 m distance (*x* = 0–20, *y* = −20 to 20, *z* = 2–20; Figure S1). The half‐hemisphere left of the array (*x* = −20 to 0) was omitted as it is identical to the right one (*x* = 0–20) due to the array's symmetry. Underground positions below the array (negative *z*‐values) were also omitted. Having calculated the TOADs for all positions and both arrays, I then generated call sequences as recorded by the array microphones, using a bat‐like chirp (linearly downward‐frequency‐modulated from 100 to 20 kHz, duration 2 ms) and a sampling rate of 5 MHz. Generated sound files were resampled to 500 kHz and analyzed with the TOADSuite (by Peter Stilz; cf., Lewanzik & Goerlitz, 2018) to obtain 3D‐positions for two values of *c* that were 2 and 5 m/s larger than the correct value used during original TOAD calculation (340 and 343 m/s).

## 3 RESULTS

**Figure 1:**
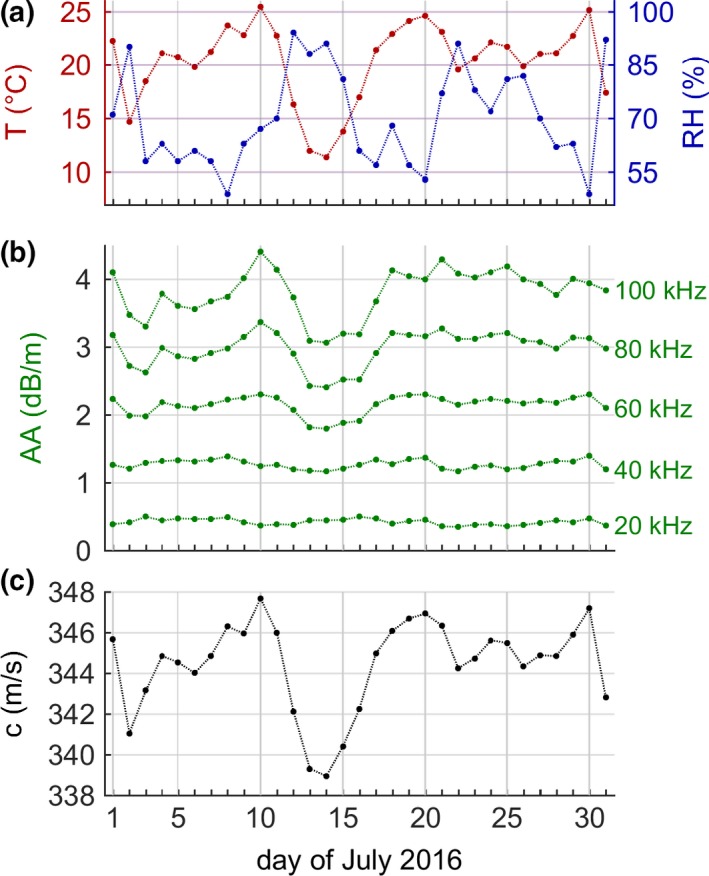
Exemplary weather and sound parameters in the first hour after sunset (at 10 p.m.) in July 2016 in Konstanz, Germany. (a) Temperature and relative humidity, (b) atmospheric attenuation for sound frequencies of 20, 40, 60, 80 and 100 kHz, (c) speed of sound in air. Note that atmospheric attenuation is mostly determined by sound frequency, but also varies with weather conditions differently for the different frequencies

Changing weather (*T*, RH; Figure 1a) can cause considerable daily variation in atmospheric attenuation (Figure 1b: AA shown at exemplary frequencies of 20, 40, 60, 80, and 100 kHz) and the speed of sound (*c*; Figure 1c). The main overall determinant of AA, however, is sound frequency, with higher frequencies always experiencing stronger and more variable AA than lower frequencies, independent of weather conditions (Figure 1b). For example, the 20‐kHz calls of a common noctule experience a roughly constant AA of about 0.5 dB/m, while AA for the 80‐kHz‐calls of a greater horseshoe bat is about 6× larger (~3 dB/m) and more variable. The AA for greater horseshoe bats decreased by about 1 dB/m after July 10th due to dropping *T* and rising RH, but remained basically constant for common noctules (except for a tiny increase). Likewise, this change in weather conditions reduced speed of sound by about 9 m/s from 348 to 339 m/s (−2.6%).

### 3.1 Atmospheric attenuation

**Figure 2:**
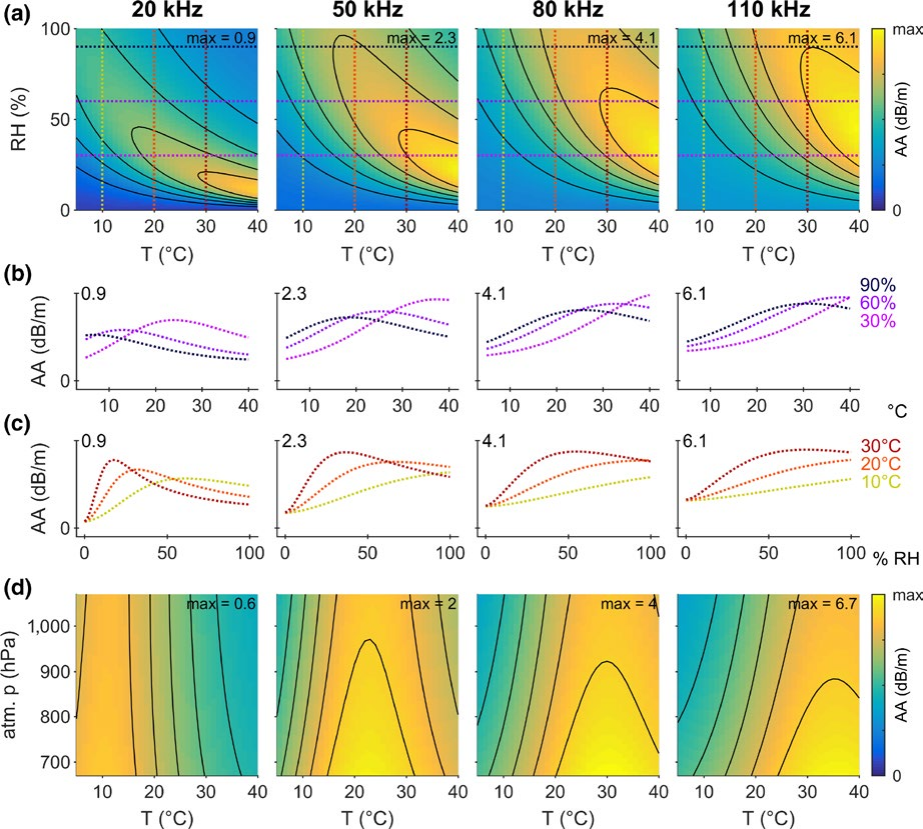
Atmospheric attenuation of sound in air (AA, separately for sound frequencies of 20, 50, 80 and 110 kHz, from left to right) varies with frequency, temperature, relative humidity and atmospheric pressure. (a) AA (color‐coded) as a function of temperature and relative humidity. Note the varying scaling of the color‐code for AA, with its maximum value given in the upper right corner of each panel. Black contour lines are drawn at equidistant and constant values of AA. Horizontal and vertical dotted colored lines indicate the cross‐sections shown in b and c. Atmospheric pressure was set to 1013.25 hPa. (b) AA as a function of temperature, shown for three relative humidities (30%, 60%, 90%, see dotted colored lines in a). Note the different scaling of the *y*‐axes matching the color code in a. (c) AA as a function of relative humidity, shown for three temperatures (10, 20, 30°C, see dotted colored lines in a). Note the different scaling of the *y*‐axes matching the color code in a. (d) AA (color‐coded) as a function of temperature and atmospheric pressure. Note the varying scaling of the color‐code for AA, with its maximum given in the upper right corner of each panel. Black contour lines are drawn at equidistant and constant values of AA. Relative humidity was set to 70%. For comparison, Figure S2 presents the same data with equal scaling of the color‐code and *y*‐axes, showing better the differences in AA with frequency instead of with *T* and RH

The AA of sound in air is determined by four factors: the sound's frequency *f* and the air's temperature *T*, relative humidity RH and pressure *p*. These four factors interact in a nonlinear fashion, resulting in a nonlinear change of AA with changing atmospheric conditions (Figure 2). AA is positively correlated with sound frequency *f*, its most important determinant, for all weather conditions, reaching maximally 0.9 dB/m for 20 kHz (within the analyzed range of weather conditions) and more than 6 dB/m for 110 kHz (Figure 2a). For each specific sound frequency, AA reaches a maximum that is located at high temperatures and, depending on frequencies, at low to medium humidities (Figure 2a). From this maximum, AA decreases slightly along a bended ridge toward lower *T* and higher RH; and reaches lowest values on either side of that ridge (toward very low and very high *T* and RH); but note the frequency‐specific differences in this pattern (Figure 2a).

Exemplary cross‐sections through these patterns as a function of *T* for three values of RH (Figure 2b) and as a function of RH for three values of *T* (Figure 2c) make these relationships more clear. With increasing temperature (Figure 2b) and relative humidity (Figure 2c), AA initially increases, reaches a frequency‐ and weather‐specific peak, and then decreases again. Note, however, that this peak may slowly move outside of the analyzed range with increasing sound frequency (e.g., cross‐sections for 30% as a function of *T*, bright purple dotted line, Figure 2b, and for 10°C as a function of RH, yellow dotted line, Figure 2c), and the slopes differ drastically for the different conditions. As examples, consider two exemplary frequencies of 20 and 50 kHz (roughly corresponding to two different bats: a common noctule with narrowband search calls with a peak frequency of about 20 kHz, and a pipistrelle bat whose search calls are more frequency‐modulated and have most energy roughly around 50 kHz). During a night with 90% RH, AA decreases for all relevant temperatures for the 20‐kHz noctule bat (dark blue dotted line, Figure 2b, first panel), but increases first until about 17°C and then decreases again for the 50‐kHz pipistrelle bat (dark blue dotted line, Figure 2b, second panel). For dryer nights with 60% RH (violet dotted line), the peak in AA is shifted by about 8–10°C to higher temperatures for both bats. Comparing the same two bats during nights with 10°C (yellow dotted line, Figure 2c), we see that the 20 kHz bat experiences maximum AA around 60% RH, while the 50 kHz bat experiences it at 100% RH. For warmer nights with 20°C (orange dotted line), this peak is shifted by about 30% points to lower humidities of about 30% and 70%, respectively. Note that the calls of many bats are frequency‐modulated, and each frequency in such a call will undergo differential atmospheric attenuation (see below and Figure 4).

Atmospheric pressure *p* also slightly influences AA. Generally, increasing *p* causes a reduction in AA, with temperature and frequency‐specific differences (Figure 2d). However, this effect is much smaller than the effects of *T* and RH, even for the large total range of *p* analyzed here (from 670 hPa at >3,000 m altitude of high‐flying *T. brasiliensis* to 1,070 hPa at −425 m altitude of the Dead Sea), which most bats will never experience.

### 3.2 Different parameters cause different amounts of variation in atmospheric attenuation

**Figure 3:**
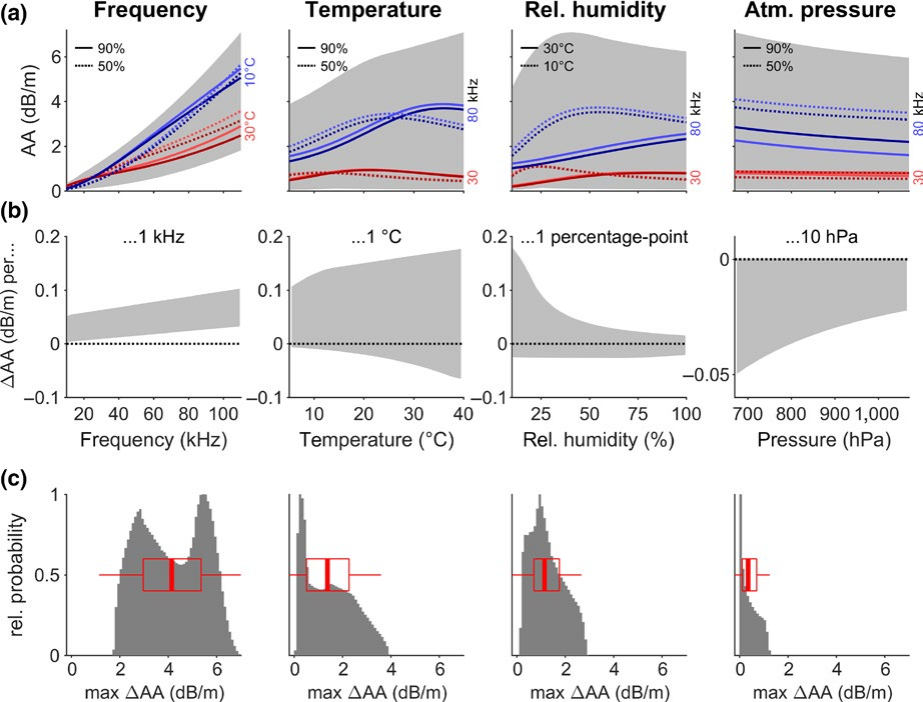
Variation in atmospheric attenuation (AA) caused by sound frequency, temperature, relative humidity, and atmospheric pressure (from left to right). (a) Total range of AA (gray area) due to variation in the remaining three parameters when the parameter on the *x*‐axis is known. The colored lines are exemplary traces of AA for exemplary values of the remaining three parameters (exemplary values are: *f *=* *30 or 80 kHz, *T *=* *10 or 30°C, RH = 50% or 90%, and *p *=* *880 or 1,060 hPa). The specific values used per line are indicated by color and line style: The parameter with the strongest effect is coded in red and blue, and the next parameter by line style. The third parameter with least effect is coded as brighter or darker color: *p* for the first three panels (bright: 880 hPa, dark: 1,060 hPa) and RH for the last panel (bright: 50%, dark: 90%). (b) Total range of AA‐change (derivative of AA, gray area) when the value on the *x*‐axis increases by a set amount (see panel title) and for all possible values of the remaining three parameters. ΔAA is always positive for increasing *f*, negative for increasing *p*, and positive and negative for increasing *T* and RH. (c) Histogram of the maximum variation in AA caused by maximum variation in one parameter, while all other parameters are kept constant, for all combinations of constant parameters. Box plots show median, quartiles and whiskers at up to 1.5 times the interquartile range beyond the quartiles. Variation in *f* causes the largest variation in AA, followed by *T* and RH, and concluded by *p* causing the smallest variation

To quantify the relative contribution of call frequency and the three weather conditions to the total variation in AA, each of the four parameters was systematically varied over its full range (Figure 3a). For each value of the focal parameter (see panel title and *x*‐axis), AA was calculated for all possible combinations of the full parameter space of the remaining three parameters (i.e., *f *=* *10–110 kHz, *T *=* *5–40°C, RH = 0%–100%, *p *=* *670–1,070 hPa), thus obtaining the range of values that AA can assume for each value of the focal parameter (gray area, Figure 3a, some exemplary curves highlighted by colored lines). As sound frequency is the strongest determinant of AA, the remaining variation is smallest when *f* is fixed (Figure 3a, left), compared to fixing the other parameters, yet still amounts to several dB/m at higher frequencies. The colored exemplary curves highlight again the nonlinear behavior of AA for increasing *T* and RH; while increasing *f* and *p* cause linearly increasing and decreasing AA, respectively.

To dissect how changes in each of the four parameters affect AA, Figure 3b presents the derivative of the curves in Figure 3a, that is, the change in AA per change in each of the parameters. Increasing call frequency always causes an increase in AA, with the exact value of ΔAA depending on the exact values of call frequency, *T*, RH and *p* and ranging between 0 and 0.1 dB/m per 1 kHz increases. Increasing air pressure always causes a decrease in AA of −0.05 to 0 dB/m per 10 hPa. In contrast, for increasing *T* and RH, the change in AA can be both positive and negative, roughly ranging between −0.05 and 0.2 dB/m for an increase of 1°C in *T* or 1% point in RH. Note, however, that changes in RH affect AA mostly for low RH below about 30%. At higher RH, which is more typical for nocturnal bat habitats, the effect of changing RH becomes smaller, meaning that AA will be mostly influenced by *T*.

Lastly, to quantify how much variation in AA is caused by each of the four parameters, three parameters were kept constant while the fourth parameter was varied over its full range to obtain the maximum variation in AA caused by variation in this focal parameter. This was repeated for all combinations of constant parameters, obtaining the maximum variation in AA caused by the focal parameter for all combinations of constant parameters (Figure 3c). Changing sound frequency from 10 to 110 kHz leads to the largest variation in AA of 2–6.5 dB/m, followed by changes in *T* and RH (0–4 and 0–2.5 dB/m, respectively). Changing atmospheric pressure over the very wide range of 670–1,070 hPa only causes variation in AA of 0–1.5 dB/m and thus can be ignored for calculating AA under most conditions.

### 3.3 Effect of weather conditions on analyzed call parameters

**Figure 4:**
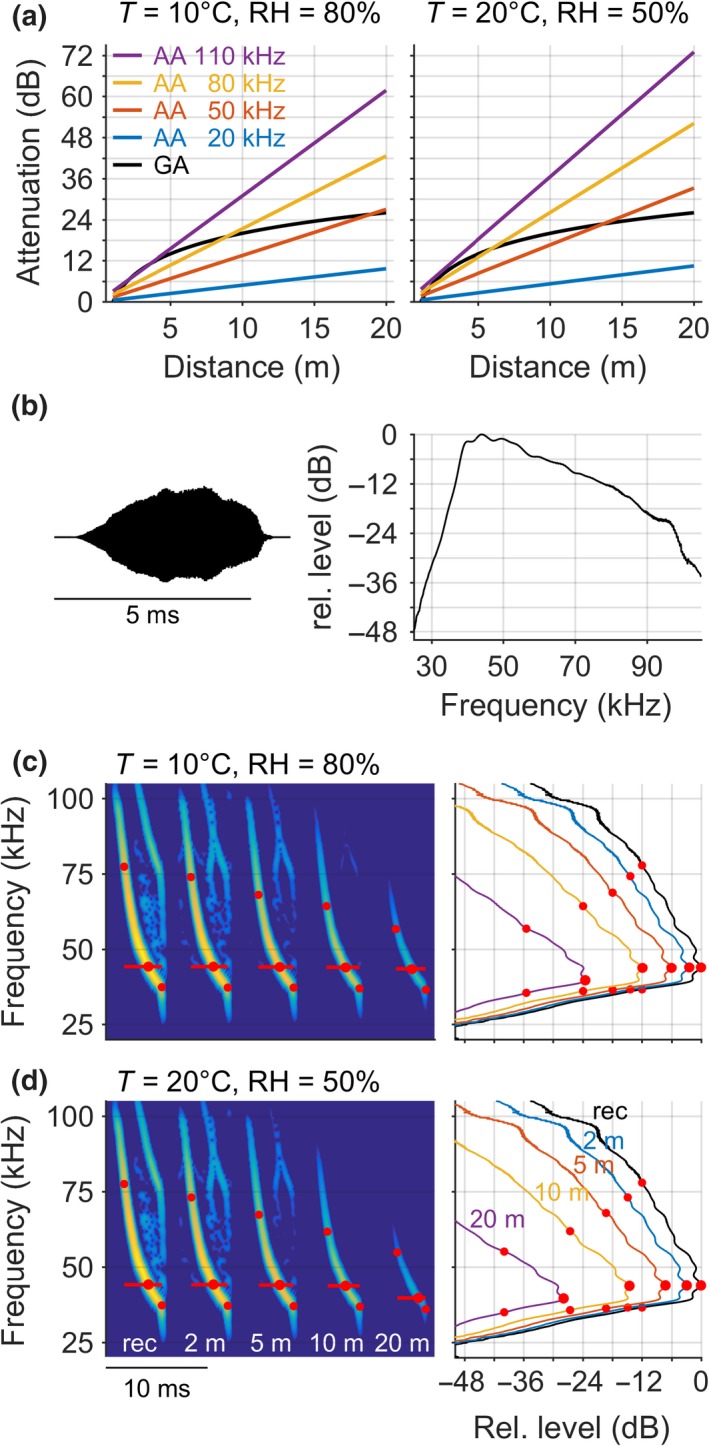
Effect of atmospheric and geometric attenuation on call level and call frequencies. (a) Geometric attenuation (black) and atmospheric attenuation for different frequencies (colored lines) as a function of increasing distance between bat and microphone, for two different weather conditions (left and right). (b) Waveform (left) and amplitude spectrum (right) of a typical frequency‐modulated call of a *Myotis* bat, recorded close to the bat at about 1 m distance. (c,d) Effect of atmospheric attenuation on measured call frequencies: spectrogram (left) and spectra (right) of the recorded call (rec) and after additional atmospheric attenuation over 2, 5, 10, and 20 m distance (colored lines), for two different weather conditions. Note how the peak frequency, the −12 dB call frequencies (red dots) and call duration (red horizontal lines, based on −12 dB threshold) are reduced with increasing distance. Geometric attenuation was disregarded. Slight differences exist in the exact peak frequencies measured in the spectrogram or spectrum, but the pattern is the same

Any recorded call will have a lower level and a different spectrum than the emitted call due to AA and geometric attenuation (GA). Geometric attenuation of bat calls usually follows spherical spreading by which the call's total energy is distributed with increasing distance over an increasingly larger spherical surface, reducing call amplitude equally for all call frequencies by 6 dB per each doubling of distance. AA and GA both increase with distance, but differently, and thus their relative contribution to the total attenuation (GA + AA) also changes with distance (Figure 4a). At short distances, total attenuation is mostly determined by GA, while at larger distances, AA becomes relatively more important as it scales per unit of distance, not per doubling of distance, particularly for higher frequencies that experience stronger AA. The exact changes in level and spectrum, however, depend on call frequency, distance, and weather conditions.

The frequency‐ and distance‐dependency of AA strongly influences the measured frequency parameters of (ultrasonic) calls, particularly for broadband calls, e.g., the frequency‐modulated calls emitted by many species of the worldwide distributed genus *Myotis*. To simulate this, we take the call of a trawling *Myotis* (*capaccinii*) recorded at about 1 m distance to the bat (Figure 4b, waveform and amplitude spectrum) and apply the frequency‐specific AA for larger distances (Figure 4c,d). With increasing distance, higher frequencies are more strongly attenuated than lower frequencies, and this effect differs between typical weather conditions (cf. Figure 1). For example, with increasing distance from 2 to 20 m, the upper −12 dB frequency (“start frequency”) is reduced by about 17 kHz (from 74 to 57 kHz) at 10°C and 80% (Figure 4c) and by about 19 kHz (from 74 to 55 kHz) at 20°C and 50% (Figure 4d), that is, by 2 kHz more than for the previous weather condition. Likewise, the frequency‐ and distance‐dependency of AA also reduces the measured peak frequency, in this example from about 44 kHz at close distances (up to 10 m) to about 40 kHz at larger distances (at 20 m), and the measured call duration, here from 3.61 ms at 2 m to 2.92 ms at 20 m (−19%, 10°C/80%) and from 3.58 ms at 2 m to 2.80 ms at 20 m (−22%, 20°C/50%; red lines in Figure 4c,d). Lastly, also note how the level of the received call is affected by weather conditions. After 20 m, the relative level at peak frequency is −24 dB for 10°C/80%, but 4 dB smaller (−28 dB) for 20°C/50%, which affects the signal‐to‐noise ratio and the call detection probability.

### 3.4 Speed of sound

**Figure 5:**
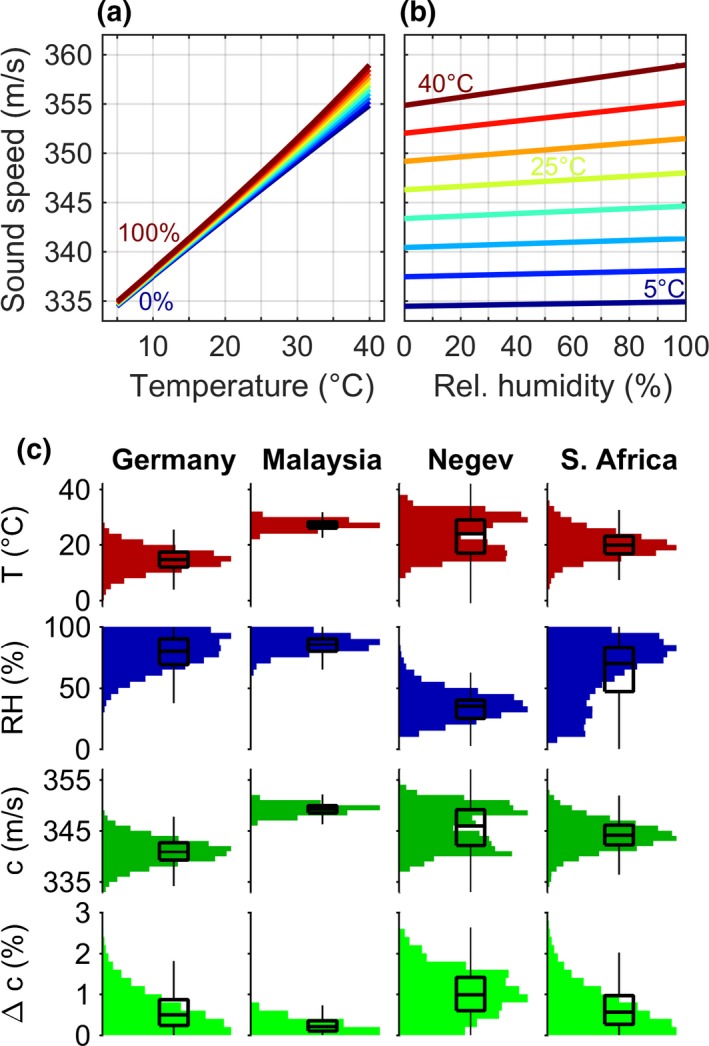
Effect of weather conditions on speed of sound in air. Speed of sound increases with temperature (a) and relative humidity (b), with a stronger effect of temperature than humidity. (c) Distribution of temperature (*T*) and relative humidity during bat foraging times in four countries with different climate, leading to different distributions in speed of sound (*c*) and relative deviation from median speed of sound (Δ*c*). Boxplots show median, quartiles and whiskers at up to 1.5× the interquartile range beyond the quartiles

Like AA, also the speed of sound (*c*) is not a constant value, but determined by temperature *T*, relative humidity RH, atmospheric pressure and the molar fraction of CO_2_. Here, we only consider *T* and RH, which are fast‐changing and have a relevant influence on *c* (Figure 5). At first approximation, *c* linearly increases with increasing *T* and RH, although the effect of *T* is much stronger than that of RH (Figure 5a,b). In more detail, *c* is a nonlinear function of *T* and RH, resulting in an interaction between both parameters. Thus, the influence of *T* on *c* becomes slightly larger at higher RH (Figure 5a), and likewise the influence of RH on *c* is stronger at higher *T* (Figure 5b). Over the full range of parameter values considered here (*T *=* *5–40°C, RH = 0%–100%), *c* varies by about 20–25 m/s (~7% total variation, ~3.5% deviation from the median), from 335 m/s at low *T* to about 355–360 m/s at high *T*. Increasing *T* by 1°C increases *c* by about 0.6 m/s. Increasing RH by 10% points causes a much smaller increase of *c* by only 0.23 m/s at 30°C and by only 0.06 m/s at 10°C.

In contrast to the full range of weather conditions of Figure 5a,b, the natural variation in weather conditions in bat habitats is smaller and variable between habitats (Figure 5c). In a temperate habitat such as Germany, *c* varies between 335 and 345 m/s, whereas *c* is larger at about 349 m/s with less variation in the tropical rain forest of Malaysia. The speed of sound in the Negev desert and South Africa lies in between those values, with larger variation in the Negev due to larger variation in *T* over the seasons. The proportional deviation of *c* from its median value also differs between the habitats, with the 75% quartile reaching values between 1% in Germany and South Africa and 1.5% in the Negev.

While this variation may seem small, it can be relevant for the increasingly used method of calculating the three‐dimensional location of sound sources based on time‐of‐arrival differences, a method that relies on the speed of sound. Using a wrong value for *c* and ignoring its variation will result in localization errors that are roughly aligned along the array‐bat‐axis in most cases. The magnitude of this error depends on the error in *c*, but also on the array geometry and distance to the array. For a symmetrical planar star‐shaped array with 60 cm intermicrophone distance, the median error is 3.6% (Q1–Q3: 1.9–6.0) of the distance to the array if *c* is assumed to be 2 m/s larger than its correct value, and 5.0% (2.9–8.3) for 5 m/s error in *c*. For a larger array of 120 cm intermicrophone distance, these errors become smaller and are 1.6% (1.1–2.7) and 3.8% (2.3–6.2).

## 4 DISCUSSION

The sounds recorded and analyzed by researchers differ from the sounds emitted by the animal due to various physical effects of the sound transmission channel. In the case of bat echolocation calls, the recorded calls have travelled some distance through the air, where it was mostly affected by geometric and atmospheric attenuation (Wahlberg & Larsen, 2017). Only if the distance to the bat and weather data are recorded (which is usually not the case for standard monitoring), can the source sound as emitted toward the direction of the microphone be reconstructed by correcting for geometric and atmospheric attenuation. In addition to these factors that are the focus of this manuscript, other factors cause additional variation in the recorded sound. First of all, variation in the relative orientation of the sonar beam axis and the microphone axis (e.g., due to head movements, beam steering or changes in flight direction) will cause significant variation in recorded call level and spectrum (Ratcliffe & Jakobsen, 2018). Only extended microphone arrays are capable of catching a sufficiently large part of the call to enable identification and reconstruction of the main sonar axis (e.g., Seibert, Koblitz, Denzinger, & Schnitzler, 2015; Surlykke et al., 2009). Further variation can be caused by flight‐induced Doppler‐shifts of sound frequency, wind and other air turbulences causing variation in sound arrival time and sound level, and temperature‐ and humidity‐gradients causing sound refraction and which cannot be considered by a single local weather measurement.

### 4.1 Atmospheric attenuation and recorded call level

In most recording situations, the distance between bat and microphone is unknown, resulting in an unknown amount of geometric and atmospheric attenuation (even if weather conditions were known). The total attenuation determines the sound level received at the microphone, and thus the distance over which sounds can be detected. This detection distance will be shorter for faint calls (e.g., those of low‐amplitude bat species, or feeding buzzes) than for loud calls (e.g., search calls), resulting in lower detection probabilities for such faint calls, which in turn might be misinterpreted as a lack of low‐amplitude bat species and of bat foraging activity. Weather‐dependent variation in AA causes additional weather‐dependent variation in the received sound level. In turn, this impacts call detection distances and thus call‐based estimates of the presence/absence, number, and foraging activity of bats.

From the viewpoint of bats, this daily, seasonal, and spatial variation in atmospheric attenuation appears to be relevant, too, as shown by frequency‐adjustments in bats that use echolocation to detect prey over long distances in the open space (Chaverri & Quiros, 2017), and bats emitting high‐frequency calls experiencing high atmospheric attenuation (e.g., Jacobs et al., 2017; Maluleke et al., 2017; Mutumi et al., 2016).

### 4.2 Atmospheric attenuation and call frequencies

The unknown amount of AA causes unknown frequency‐specific changes of the recorded call. Measured call frequencies thus vary not only when bats change call parameters (Kalko & Schnitzler, 1993), but also simply when the distance between bat and microphone changes. This effect is particularly pronounced for high frequencies (i.e., typically the start frequencies of calls) which experience stronger AA than low frequencies. Together with other unknown factors, such as scanning movements of the bat and variation in the relative orientation between call direction and microphone axis, this causes large variation in measured call frequencies between subsequent calls and between different published studies (Thomas, Bell, & Fenton, 1987). This variation is larger for broadband frequency‐modulated calls (e.g., *Myotis* bats, frequency‐modulated part of the call of high‐duty cycle bats such as horseshoe bats) than narrowband calls (e.g., noctule bats, constant frequency part of the call of high‐duty cycle bats). Presenting bat call frequencies to an accuracy of less than 1 kHz is for this reason discouraged (in addition to further limitations in frequency resolution of the analysis algorithm, e.g., the FFT size). Only methods that localize bats in space and compensate for the distance‐ and frequency‐specific AA for each recorded call may allow for higher precision (e.g., Seibert et al., 2015; Surlykke et al., 2009).

This unknown (and thus: noncompensable) variation in frequency parameters, in addition to the naturally existing variability and overlap in call parameters between species, may limit our capability of identifying bat species based on call recordings (Russo & Voigt, 2016; Rydell et al., 2017). This limitation is worse for species‐rich areas and for sympatric species with similar call designs (e.g., many *Myotis* species). In contrast, in areas with less species and/or for species with unique call designs this is less or not problematic. Where limitations exist, these still seem to apply more to automatic species identification than to manual identification by experienced bat researchers, yet both methods are not error‐free and require extensive training/experience and careful validation (Fritsch & Bruckner, 2014).

### 4.3 Speed of sound

The variation in weather conditions sets a lower environmental limit for the ranging accuracy of echolocation. To convert perceived echo delay into object distance, bats require some internal representation of speed of sound. A mismatch between the internal and real speed of sound will cause errors in the perceived object distance. Varying weather conditions cause variations in the speed of up to 3% leading to distance measurement errors of up to 3% if bats cannot adjust their internal representation. As this is a proportional error, however, the absolute error becomes smaller as the bat approaches an object, from 3 cm at 1 m object distance to only 3 mm at 10 cm object distance. Therefore, I argue that ranging errors caused by unknown variation in speed of sound are negligible for bats, even for the most demanding tasks of pinpointing insects or landing on small twigs.

However, the unknown variation in speed of sound has another consequence on echo delay perception. An average variation in speed of sound of just ±0.5% (Figure 5c) causes an average variation in echo delay of about 30 μs at 1 m object distance. This environmental noise is much larger than the submicrosecond delay accuracy proposed for the bat *Eptesicus fuscus* (e.g., Moss & Schnitzler, 1989; Simmons, 1979; Simmons, Ferragamo, Moss, Stevenson, & Altes, 1990), yet matches the measured free‐flight accuracy of 20–25 μs in the species *Glossophaga soricina* (Goerlitz, Geberl et al., 2010). Even if bats had submicrosecond delay accuracy, the amount of environmental noise would prohibit a corresponding submillimeter accuracy in absolute target ranging. In contrast, the measurement of *relative* differences in echo‐delay and object‐range between two targets, or a single moving target over time, would not be impaired.

Most bioacoustic measurements will be unaffected by variation in speed of sound, except for the acoustic localization of animals in 3D‐space based on call time‐of‐arrival differences (e.g., Fujioka et al., 2016; Goerlitz, ter Hofstede et al., 2010; Hügel et al., 2017; Seibert et al., 2015; Surlykke et al., 2009). Localization errors due to imprecise values for *c* are mostly oriented along the array‐bat‐axis (i.e., affecting the estimate of the bat's distance to the array), which is most susceptible to localization errors as comparatively large changes in distance generate only comparatively small and nonresolvable changes in time‐of‐arrival differences—*vice versa*, comparatively small changes in time‐of‐arrival differences cause comparatively large errors in distance. Although this error is rather small (Q3: 8.3%) even for large mismatches in *c* (5 m/s) and small arrays (60 cm intermicrophone distance), additional error sources such as low signal‐to‐noise ratio, Doppler shifts or variation in call envelope will impede localization accuracy. Thus, to reduce sound‐speed based errors, acoustic tracking should always consider local weather conditions (e.g., Goerlitz, ter Hofstede et al., 2010; Lewanzik & Goerlitz, 2018).

## 5 CONCLUSION

Weather conditions determine two physical sound parameters that are crucial for bats and researchers: the atmospheric attenuation and the speed of sound in air. Weather‐ and frequency‐dependent variation in atmospheric attenuation causes variation in the received sound level and call spectra, and thus variation in detection distances and call frequencies. In turn, this may limit call‐based measurements of the presence/absence and number of bats and bat species identification, depending on the amount of local weather variation and the similarity in call design of the local bat assemblage.

## CONFLICT OF INTEREST

None declared.

## AUTHOR CONTRIBUTIONS

HRG conducted all work presented in this article, including research design, data analysis, figure preparation and writing.

## DATA ACCESSIBILITY

Data was archived in DRYAD (https://doi.org/10.5061/dryad.k5d3280).
