# Supplementary material for: Weather conditions determine attenuation and speed of sound: Environmental limitations for monitoring and analyzing bat echolocation
Source: Ecol Evol. 2018 Apr 24;8(10):5090–100. doi: 10.1002/ece3.4088 (PMC5980448; doi:10.1002/ece3.4088)
Supplement: Supplementary file 1 [file ECE3-8-5090-s001.pdf]

Weather conditions determine attenuation and speed of sound: environmental limitations for monitoring and analysing bat echolocation

Holger R. Goerlitz

Acoustic and Functional Ecology Group, Max Planck Institute for Ornithology, Seewiesen, Germany

hgoerlitz@orn.mpg.de

EQUATIONS

Atmospheric attenuation of sound in air (ISO standard #9613-1, ISO, 1993)

```
%% required parameters:
sound frequency F (Hz)
temperature T (°C)
relative humidity RH (%)
atmospheric pressure p (Pa)

%% convert parameters:
Ta = T+273.15       % convert to Kelvin
Tr = Ta/293.15      % convert to relative air temperature (re 20°C)
pr = p/101325       % convert to relative pressure


% Saturation Concentration of water vapour.
C = 4.6151 - 6.8346 * ((273.16/Ta)^1.261)

% percentage molar concentration of water vapour:
h = RH * 10^C / pr

% relaxation frequencies associated with the vibration of oxygen and
nitrogen:
frO = pr * (24 + 4.04e4*h*(0.02+h)/(0.391+h))
frN = pr * (Tr^(-0.5)) * (9 + 280*h*exp(-4.17*((Tr^(-1/3))-1)))

% attenuation coefficient alpha (Np/(m*atm)):
alpha = f*f * ...
    ( 1.84e-11*(1/pr)*sqrt(Tr) + ...
    (Tr^(-2.5)) * ( 0.01275*(exp(-2239.1/Ta)*1/(frO+f*f/frO)) + ...
    0.1068*(exp(-3352/Ta) * 1/(frN+f*f/frN)) ) )

% convert alpha to dB (lg(x/x0)) from Neper (ln(x/x0)):
AA = 8.686 * alpha;         % attenuation coefficient (dB/m)
```

Speed of sound in air (Cramer, 1993)

```
%% required input parameters:
temperature T (°C)
relative humidity RH (%)
atmospheric pressure p (Pa)
molar fraction of CO2 in the atmosphere xc (ppm)


%% coefficients (Cramer 1993):
a0 = 331.5024
a1 = 0.603055
a2 = -0.000528
a3 = 51.471935
a4 = 0.1495874
a5 = -0.000782
a6 = -1.82e-7
a7 = 3.73e-8
a8 = -2.93e-10
a9 = -85.20931
a10 = -0.228525
a11 = 5.91e-5
a12 = -2.835149
a13 = -2.15e-13
a14 = 29.17976
a15 = 0.000486


%% convert molar fraction of CO2 from ppm into fraction:
xc = xc / 1e6


%% Calculating the mole fraction of water vapour in air based on the
relative humidity, according to Giacomo (1982), eq. 19:

% Enhancement factor:
f = 1.00062 + 3.14e-8*p + 5.6e-7*T^2;

% Saturation vapour pressure of water vapour in air:
Ta = T + 273.15    % convert to Kelvin
psv = exp(1.2811805e-5*Ta.^2 - 1.9509874e-2*Ta + 34.04926034 -
6.3536311e3./Ta)

% water vapour mole fraction:
RHfrac = RH/100;    % relative humidity expressed as fraction
xw = RHfrac * f * psv / p;


%% Calculate speed of sound (m/s; Cramer 1993):
c = a0 +...
    a1*T +...
    a2*T^2 +...
    (a3 + a4*T + a5*T^2) * xw +...
    (a6 + a7*T + a8*T^2) * p +...
    (a9 + a10*T + a11*T^2) * xc +...
    a12*xw^2 +...
    a13*p^2 +...
    a14*xc^2 +...
    a15*xw*p*xc;
```

SUPPORTING FIGURE

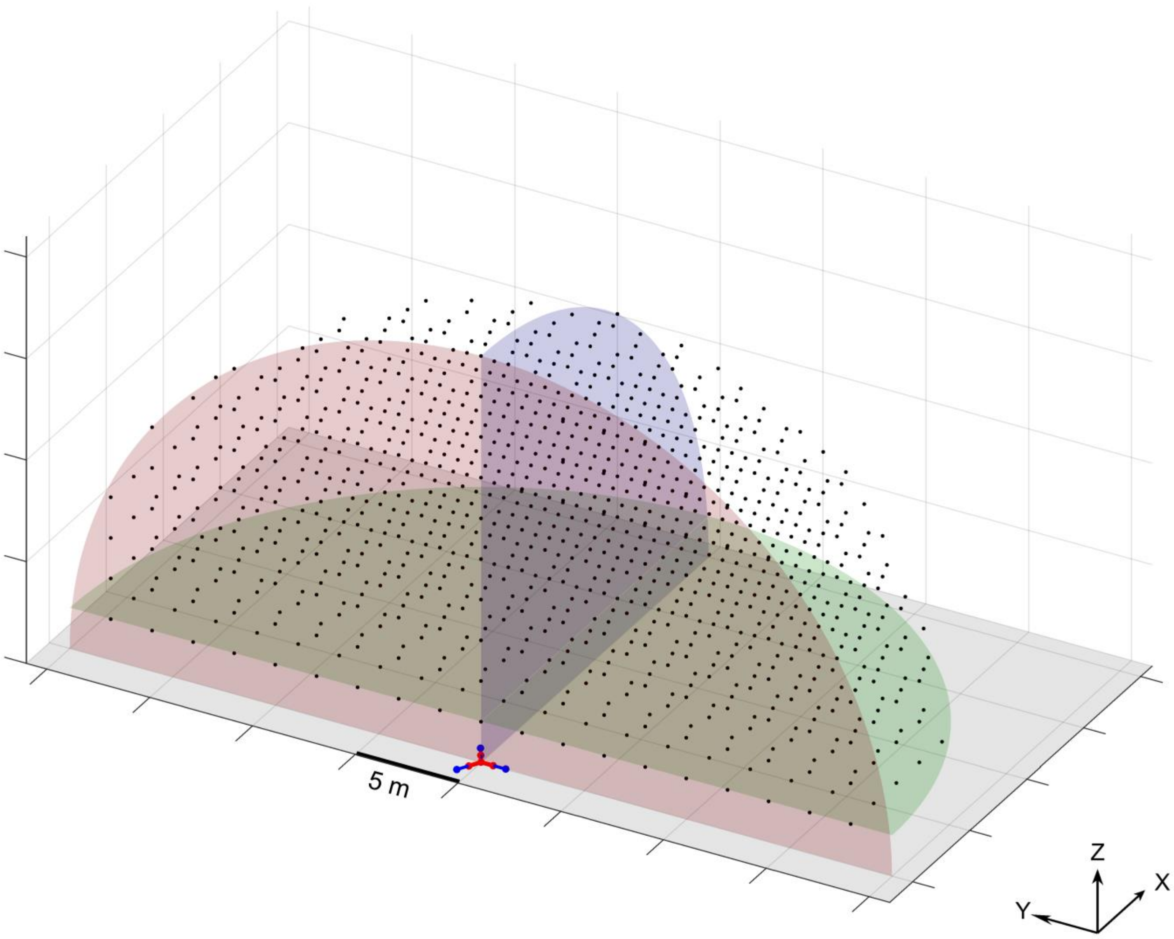

Figure S1: Positions of the microphone arrays and sound sources which were used to calculate the effect of variation in speed of sound on the localization accuracy of acoustic tracking.

The two symmetrical planar star-shaped four-microphone arrays are shown in red and blue (60 cm and 120 cm inter-microphone distance, respectively). The sound source positions are shown as black dots. They are arranged in a grid with 2 m spacing up to a distance of 20 m to the array and fill the volume above (z > 0) and to the right of (x >= 0) the array. Coloured areas indicate three exemplary planes of sound sources: the vertical plane above the array in the YZ-plane (red), the vertical plane above the array in the XZ-plane (blue) and the horizontal plane at 2 m height above the array (parallel to the XY-plane).

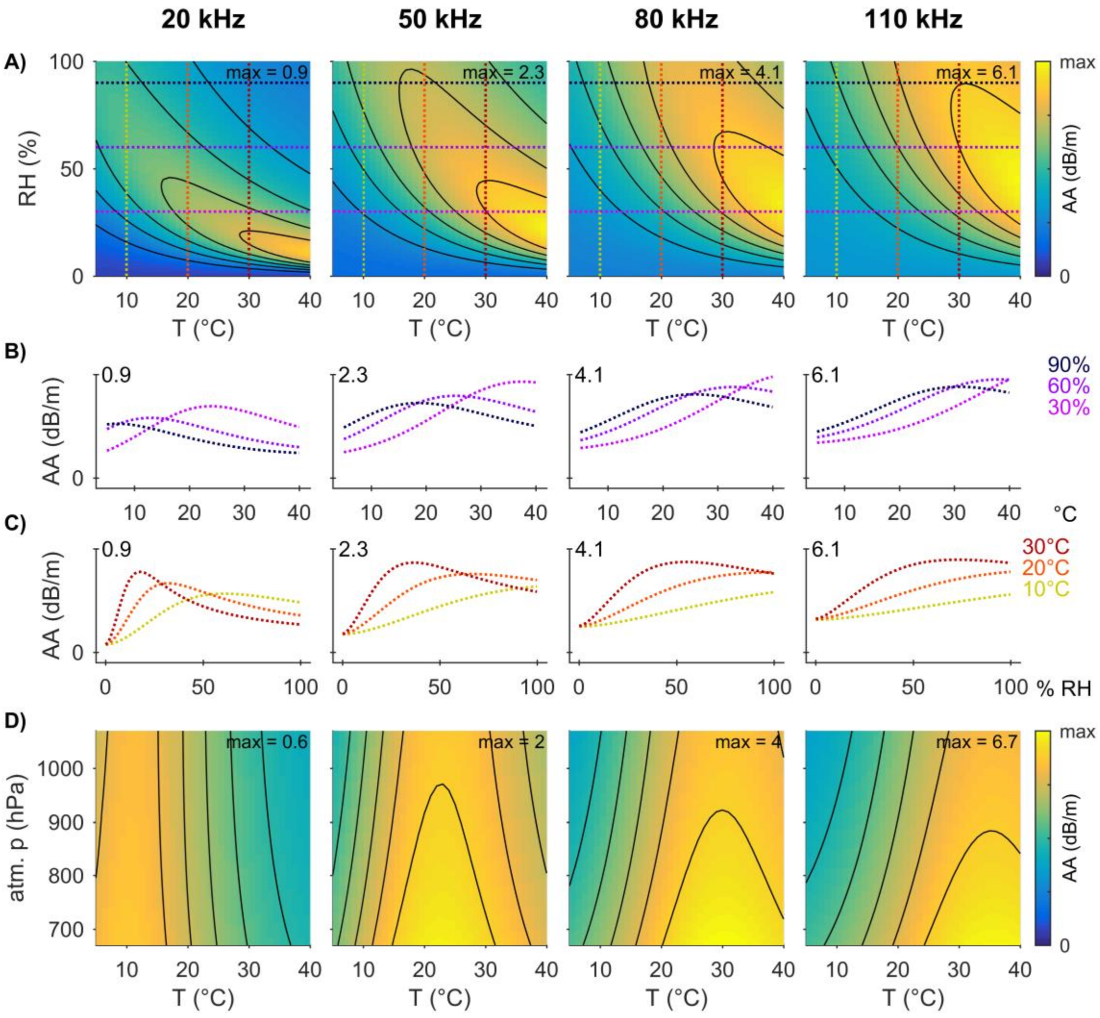

Fig. S2: Atmospheric attenuation of sound in air (*AA*, separately for sound frequencies of 20, 50, 80 and 110 kHz, from left to right) varies with frequency, temperature, relative humidity and atmospheric pressure.

A) *AA* (colour-coded) as a function of temperature and relative humidity. Black contour lines are drawn at equidistant and constant values of *AA*. Horizontal and vertical dotted coloured lines indicate the cross-sections shown in **B** and **C**. Atmospheric pressure was set to 1013.25 hPa.

B) *AA* as a function of temperature, shown for three relative humidities (30, 60, 90%, see dotted coloured lines in **A**).

C) *AA* as a function of relative humidity, shown for three temperatures (10, 20, 30°C, see dotted coloured lines in **A**).

D) *AA* (colour-coded) as a function of temperature and atmospheric pressure. Black contour lines are drawn at equidistant and constant values of *AA*. Relative humidity was set to 70%.

This figure shows the same data as **Fig. 2**, yet with equal scaling of the colour-code and y-axes.

REFERENCES

Cramer, O. 1993. The variation of the specific heat ratio and the speed of sound in air with temperature, pressure, humidity, and CO_2 concentration. *Journal of the Acoustical Society of America,* 93, 2510-2516.

International Organisation for Standardization 1993: ISO 9613-1: Acoustics - Attenuation of sound during propagation outdoors – Part 1: Calculation of the absorption of sound by the atmosphere. American National Standard Institute, New York.

Giacomo, P. 1982. Equation for the determination of the density of moist air (1981). *Metrologia* 18, 33-40.
